# Viviparous mothers impose stronger glucocorticoid‐mediated maternal stress effects on their offspring than oviparous mothers

**DOI:** 10.1002/ece3.8360

**Published:** 2021-11-29

**Authors:** Kirsty J. MacLeod, Geoffrey M. While, Tobias Uller

**Affiliations:** ^1^ Department of Biology Lund University Lund Sweden; ^2^ School of Natural Sciences University of Tasmania Hobart Tas. Australia

**Keywords:** glucocorticoids, maternal effects, maternal stress, meta‐analysis, viviparity

## Abstract

Maternal stress during gestation has the potential to affect offspring development via changes in maternal physiology, such as increases in circulating levels of glucocorticoid hormones that are typical after exposure to a stressor. While the effects of elevated maternal glucocorticoids on offspring phenotype (i.e., “glucocorticoid‐mediated maternal effects”) have been relatively well established in laboratory studies, it remains poorly understood how strong and consistent such effects are in natural populations. Using a meta‐analysis of studies of wild mammals, birds, and reptiles, we investigate the evidence for effects of elevated maternal glucocorticoids on offspring phenotype and investigate key moderators that might influence the strength and direction of these effects. In particular, we investigate the potential importance of reproductive mode (viviparity vs. oviparity). We show that glucocorticoid‐mediated maternal effects are stronger, and likely more deleterious, in mammals and viviparous squamate reptiles compared with birds, turtles, and oviparous squamates. No other moderators (timing and type of manipulation, age at offspring measurement, or type of trait measured) were significant predictors of the strength or direction of the phenotypic effects on offspring. These results provide evidence that the evolution of a prolonged physiological association between embryo and mother sets the stage for maladaptive, or adaptive, prenatal stress effects in vertebrates driven by glucocorticoid elevation.

## Graphical abstract

Maternal “stress” effects mediated by glucocorticoid hormones have been the focus of many studies. We synthesize this information in a meta‐analytical framework to investigate overall patterns and the importance of key moderators, in particular reproductive mode. We show that glucocorticoid‐mediated maternal effects are generally stronger and more negative in viviparous species, suggesting that prolonged fetal–maternal interaction has additional evolutionary consequences.

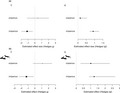

## 1 INTRODUCTION

Mothers can affect the phenotype of their offspring through many non‐genetic means (Badyaev & Uller, 2009). Such maternal effects explain, on average, 11% of the phenotypic variance observed in natural populations (Moore et al., 2019). This can make maternal effects important drivers of ecological and evolutionary dynamics, for example, by causing lags in population growth, biasing the distribution of phenotypes available for selection, or enabling adaptive adjustment of offspring phenotype to local conditions (Räsänen & Kruuk, 2007; Uller, 2008, 2012; Wolf & Wade, 2016). Accordingly, identifying general patterns of maternal effects in natural populations is of substantial interest to ecology, evolution, and conservation biology.

Maternal exposure during offspring development to stimuli that elicit a stress response is one potential source of maternal effects (Love et al., 2013; Meaney et al., 2007). The physiological response to stress exposure in amniotes (mammals, birds, and reptiles) is highly conserved (Monaghan, 2014; Taborsky et al., 2021), and as a result, maternal stress exposes offspring to elevated glucocorticoids (metabolic hormones upregulated in response to stressors as part of the hypothalamic–pituitary–adrenal axis [HPA] response [Wingfield et al., 1998]). For example, elevated stress/glucocorticoids in avian mothers result in increased deposition of glucocorticoids in the yolks of their eggs (Hayward & Wingfield, 2004). In live‐bearing species, such as mammals, circulating glucocorticoids can cross the placenta to the developing embryo (Matthews, 2007). Increased exposure to glucocorticoids in the prenatal environment can influence numerous aspects of offspring phenotypic development, including endocrine physiology, behavior, and metabolism (Boersma & Tamashiro, 2015; Sapolsky et al., 2000; Sheriff et al., 2017). Such effects, which we will term “glucocorticoid‐mediated maternal effects,” have been suggested to be an important contributor to population dynamics and a cause of selection on both mothers and offspring (reviewed in Sheriff et al., 2017; Sheriff & Love, 2013).

While glucocorticoid‐mediated maternal effects on phenotype and fitness of offspring have been relatively well established in laboratory studies (Seckl, 2004), it remains poorly understood how strong and consistent these effects are in natural populations (Sheriff & Love, 2013; Taborsky et al., 2021). Furthermore, the functional consequences of maternal stress, including downstream effects on offspring via glucocorticoid and other pathways, remain controversial (Harris, 2020). While laboratory studies tend to interpret offspring responses to maternal stress/glucocorticoids as negative for *both* the mothers and the offspring's fitness, these may be negative to offspring only (Marshall & Uller, 2007), or even represent active, adaptive, adjustment of morphology, physiology, and behavior to local conditions (Sheriff & Love, 2013). Establishing if there are general patterns in the strength and direction of glucocorticoid‐mediated maternal effects in nature and identifying the ecological or life history characteristics that moderate those effects are the first steps in promoting a more informed interpretation of their evolutionary origin and population consequences. In line with this, quantitative meta‐analyses have arguably been instrumental for interpreting the ecological and evolutionary consequences of maternal effects more generally (Moore et al., 2019; Sánchez‐Tójar, Lagisz, et al., 2020; Uller et al., 2013; Yin et al., 2019).

One life history characteristic that has the potential to have particularly important implications for the scope of both adaptive and maladaptive maternal stress effects, including those mediated by glucocorticoids, is reproductive mode (e.g., oviparity vs viviparity). In viviparous species (where embryonic development takes place inside the body of the parent, such as the mammals), embryos typically exhibit a much more prolonged physiological association with their mother than the embryos of oviparous species (where embryos develop in and hatch from externally deposited eggs, such as the birds) (Blackburn, 1999). However, the extent to which a prolonged fetal–maternal connection promotes maternal effects, and the effects of prenatal glucocorticoids specifically, is not straightforward. On the one hand, the placenta can buffer offspring from increases in maternal hormones. For example, 11β‐hydroxysteroid dehydrogenase type 2 (11β‐HSD2), an enzyme that converts glucocorticoids to inert forms (Seckl, 2004), is an important regulator of glucocorticoid bioavailability in fetal tissues across the vertebrates, but high expression in placental tissue is an important additional buffer beyond endogenous fetal production in viviparous species (Wyrwoll et al., 2011). On the other hand, adversity during the prenatal period can result in downregulation of these enzymes (Jensen Peña et al., 2012), resulting in increased embryonic exposure in times of maternal stress relative to normal levels (i.e., where up to 10%–20% of maternal glucocorticoids can cross to the fetus in placental mammals; Meaney et al., 2007).

While there is a significant potential for prolonged and considerable glucocorticoid transfer during development in viviparous species, the levels of maternal glucocorticoids deposited in the yolk of oviparous reptiles and birds are often low (e.g., relative to other steroid hormones; Groothuis & Schwabl, 2008) and can be metabolized in early development (Carter et al., 2018; Vassallo et al., 2014). Yet, recent research suggests that even metabolized forms of maternally derived glucocorticoids may influence offspring neural development and thus long‐term behavioral traits, via the neurosteroid pathway (Mouton & Duckworth, 2021). Despite these clear differences in the possible pathways for glucocorticoid‐mediated maternal effects between viviparous and oviparous species, there has been no formal assessment of the extent to which these result in quantifiable differences in the wild. However, predictions about the relative strength and general direction of these effects, from which we might make some inference about their benefits or costs, remain to be explicitly tested.

To further our understanding of the effects of maternally derived glucocorticoids in amniotes, we conducted a meta‐analysis of studies investigating maternal stress effects mediated by glucocorticoid elevation on offspring phenotype in wild mammals, birds, and reptiles. We first assess the overall effects of maternally elevated glucocorticoids on offspring traits. We then investigate the potential influence of a number of key moderators (Table 1). Specifically, we quantify the strength of these effects across reproductive modes. We first do this across broad taxonomic boundaries (birds, mammals, turtles, squamate reptiles) and then leverage the considerable variation in reproductive mode within squamate reptiles (Blackburn, 2006) to disentangle the effects of reproductive mode from broader phylogenetic effects. We also test whether several other life history traits and experimental methodology influence the magnitude and direction of glucocorticoid‐mediated maternal effects: including the timing and type of maternal/prenatal manipulation; age at offspring measurement; and the type of trait measured. The biological rationale for these moderators is summarized in Table 1.

**TABLE 1:** Moderators included in global models testing predictions about glucocorticoid‐mediated maternal effects on offspring phenotype

Moderator	Levels	Prediction	References
Reproductive mode	Oviparous; viviparous	Effects—both positive and negative—should be stronger in viviparous species due to prolonged period of feto‐maternal interaction	Meaney et al. (2007), Schatten and Constantinescu (2008)
Timing of maternal manipulation	Early development; late development; throughout development	Effects should be stronger when treatment includes early development due to high embryonic sensitivity at this time	Berghänel et al. (2017)
Manipulation type	Ecological stressor; HPA axis manipulation	Direct manipulations of the HPA axis may produce stronger effects than natural stressors	Sopinka et al. (2015), Schoenle et al. (2021)
Age at offspring measurement	At/around birth (perinatal); juvenile; maturity	Phenotypic effects of prenatal glucocorticoid exposure should be strongest early in ontogeny	Moore et al. (2019), Yin et al. (2019), Wilson and Réale (2006)
Trait category	Size/mass; physiology; behavior/performance; stress response; survival	Effects should be stronger in traits with high levels of plasticity to prevailing conditions (size/mass) or susceptibility to change via the neurosteroid pathway (behavior/performance), or in traits relevant to “environmental matching” (physiology, in particular stress response). Effects on size/mass and survival more likely to be negative.	Kuijper & Hoyle (2015), Berghänel et al. (2017), Mouton and Duckworth (2021)

## 2 METHODS

We conducted a comprehensive literature search between February 8 and March 4, 2019, for studies in which amniote mothers (reptiles, mammals, birds) were subjected to an experimental treatment designed to test the effects of glucocorticoid‐mediated maternal effects. We followed the PRISMA statement checklist (Preferred Reporting Items for Systematic Reviews and Meta‐Analyses (Moher et al., 2009); Appendix 1). We searched for papers on ISI Web of Science and Scopus (all collections, inclusive of year ranges) using the following keyword combinations: transgenerational glucocorticoid/corti*; anticipatory maternal effect; environmental matching; prenatal stress/glucocorticoid/corti*; transgenerational stress mammal/bird/reptile; maternal corti* bird/mammal/reptile; maternal stress wild rodent; maternal glucocorti*; maternal stress effect mammal ‐human ‐rat ‐domestic ‐depression; and maternal stress effect bird/reptile. We used keyword combinations rather than Boolean search strings to increase redundancy and to maximize the number of records returned. We also made use of the references in major reviews (Berghänel et al., 2017; Breuner, 2008; Eyck et al., 2019; Henriksen et al., 2011; Podmokła et al., 2018; Schoech et al., 2012; Thayer et al., 2018; Veru et al., 2014). We repeated the search protocol in October 2020 to include papers published in 2019 and 2020.

Using the web application Rayyan (Ouzzani et al., 2016), duplicated studies were removed, and unique abstracts were then scanned by the lead author for indications that the study fulfilled our basic criteria, which were as follows: (1) eligible studies tested the effects of elevated “stress” during development by measuring offspring traits; (2) maternal stress treatments included both direct manipulations of maternal stress via physiological manipulations, such as glucocorticoid administration, and indirect manipulations of maternal stress via manipulation of environmental stressors, such as predation cues, as well as studies in which embryos were directly exposed to signals of maternal stress, such as manipulations of yolk hormones, designed to approximate or mimic natural maternal transfer, *but*; (3) studies in which environmental stressors were used instead of direct hormone manipulation were only included if the treatment was or had previously been shown to significantly elevate maternal glucocorticoids (i.e., by also testing glucocorticoid effects or downstream activation of the HPA axis) either in the same study or in a different study of the same species referenced in the original study; (4) only studies manipulating stress exposure within a breeding season were considered (i.e., we included studies in which prenatal stress was manipulated during mating/fertilization to gestation and egg laying, but did not include studies where the effects of early life stress were tested on later reproduction); and (5) given that our primary aim was to understand how maternally derived glucocorticoids mediated offspring development in the wild, we excluded studies that utilized domesticated or captive populations (>1 generation bred in captivity), and we did not include any studies conducted on humans. While maternal experience of stress is not limited to glucocorticoid elevation, and therefore, there is the potential for maternal stress effects via other routes (i.e., glucocorticoids and "stress" are not synonymous: MacDougall‐Shackleton et al., 2019), we focus specifically on glucocorticoid‐mediated maternal stress effects for three reasons. First, glucocorticoid elevation is an extremely well‐studied marker of maternal and prenatal stress in the ecological literature (Bonier et al., 2017), resulting in a large number of studies available with comparable experimental designs. Second, by focusing on one potential mechanistic route by which maternal stress effects might influence offspring phenotype, we hope to draw conservative and meaningful conclusions across groups that may differ in other aspects of the stress response. Third, how steroid hormones such as glucocorticoids are transferred to offspring is a key difference between viviparous and oviparous species, and thus represents a major mechanism behind an increased scope for both positive and negative phenotypic effects of maternal stress on the next generation in viviparous species.

Following the above refinement, we ended up with a final list of 49 eligible papers (see PRISMA in Appendix 1 for full details). To calculate effect sizes, we extracted treatment and control group means, sample sizes, and errors (SD) for each trait measured in offspring from each of these papers. The traits measured in offspring were highly heterogeneous across studies. We predicted that the strength and direction of glucocorticoid‐mediated maternal effects might differ depending on the type of trait measured (Table 1). To test this, we assigned each trait to a broad grouping category (detailed in Appendix 2); namely, traits could be grouped as physiological (e.g., hematocrit, lymphocyte count, *N* = 70 traits); related to behavior or performance (e.g., righting response time, time basking, *N* = 81 traits); size and mass measurements (*N* = 155 traits); related to the stress response (e.g., baseline glucocorticoid levels, *N* = 41 traits); and lastly, measures of survival (*N* = 15 traits).

We also extracted study information, including information on each moderator (Table 1). This included whether the study species was oviparous or viviparous (reproductive mode), the category of the trait measured (as described above), and manipulation type (as per the above criteria), as well as information on age at offspring measurement and timing of maternal manipulation. Age at offspring measurement was binned into three categories: perinatal (at or within 3 days of birth), juvenile (before maturity), and maturity. To attempt to encompass the range of species included (and in particular, variation in reproductive mode) in a broadly comparable way, we binned timing of maternal treatment into three categories: manipulations carried out in early gestation, or in the pre‐egg‐laying or egg‐laying period were designated as “early development”; those carried out in mid‐ to late gestation or during incubation were designated as “late development”; and those that continued through multiple periods were designated as “throughout development.” Where the above values were not presented in the text, data were generated by either extracting data from figures using the R package *metaDigitise* (Pick et al., 2018), extracting data from supplementary material, or contacting the authors directly. Inability to obtain data resulted in the removal of one study (one effect size) and five effect sizes from two other studies. In cases where there were multiple controls (e.g., an unmanipulated group, and a standardized control such as an oil vehicle treatment in experiments where glucocorticoids were elevated using an oil suspension applied cutaneously), the standardized control was used (*N* = 6 studies). Our search keywords and criteria targeted studies testing maternal stress (i.e., not paternal or biparental); however, in a very small number of studies (*N* = 2) experimental manipulations targeted nests or groups rather than individuals so it is possible that fathers also experienced increased stress, which may have also influenced offspring traits (Chan et al., 2018).

The final dataset comprised 394 individual effect sizes, from 23 species, and 48 eligible studies. The number of effect sizes was similar for birds (6 species, *N* = 143 traits), mammals (6 species, *N* = 110 traits), and reptiles (11 species, 2 turtles, 1 snake, and 8 lizards; *N* = 141 traits). All turtles are oviparous, but there was a roughly equal split of oviparous species (4, *N* = 31 traits) and viviparous species (5, *N* = 87 traits) in squamate reptiles. Together, this resulted in data for a total of 197 traits across 11 viviparous species and 197 traits across 12 oviparous species being included in our dataset.

### 2.1 Statistical analysis

To determine maternal stress effects on offspring traits, we calculated the effect size using Hedges’ *g* (i.e., bias‐corrected standardized mean difference; Nakagawa & Cuthill, 2007) and associated sampling variance for each trait in our dataset (*N* = 394 traits) using the package *metafor* (Viechtbauer, 2010) in software R (version 3.5.2.; R Core Team, 2018). It has been suggested that increased phenotypic variation (which influences the potential for selection based on differential fitness) is expected under stress (Hoffmann & Hercus, 2000; Sánchez‐Tójar, Moran, et al., 2020). We therefore also calculated the log coefficient of variation ratio (lnCVR; Nakagawa et al., 2015) and associated sampling variance to test for differences in variance between the compared groups ( Sánchez‐Tójar, Moran, et al., 2020). We used the *metafor* package (version 3.0.2) for all further analysis unless specified otherwise. To control for phylogenetic effects, we constructed a phylogenetic tree specific to our dataset using a synthetic super‐tree from the Open Tree of Life database (Hinchliff et al., 2015), accessed and pruned through the R package *rotl* (version 3.0.10; Michonneau et al., 2016; Appendix 3).

Effect size signs were systematically changed to allow some biological interpretation in terms of “positive” vs “negative” effects. For example, an increase in time taken to learn a simple task or perform a righting response represents positive effect sizes with “negative” effects, so here the effect size sign was changed to negative (effect sizes changed for *N* = 44 traits; all traits, with effect size signs, presented in Appendix 2). Where there was ambiguity about the assumed benefit of an increase or decrease in a trait value, effect size sign was left unchanged (e.g., an increase in metabolic rate, hormone titers, or activity rate may be “positive” in a numerical sense—but not necessarily biologically “positive” in the sense of increasing offspring fitness). This means that, across all traits, negative values mean that offspring from mothers with elevated glucocorticoids have lower values—for example, smaller, slower, less active, with reduced metabolic rate or growth, relative to control offspring. While this is consistent with the authors’ interpretation of the effects on offspring fitness, we acknowledge that whether effects on trait values are truly adaptive or maladaptive is considerably more nuanced and likely to depend strongly on context (Marshall & Uller, 2007). To be able to draw more robust conclusions about patterns seen in the whole dataset, we additionally created a more conservative subset of the data where traits were less ambiguous in whether an increase or decrease represented a positive or negative outcome based on life history theory—for example, in mass, size, or survival measures (all traits included in this subset are presented in Appendix 2).

#### 2.1.1 Overall effects

To test the strength of glucocorticoid‐mediated maternal effects, we first constructed a random‐effects model with Hedges’ *g* as the response variable. We included study ID as a random effect to control for non‐independence among estimates from the same study and an observation‐level random‐effects term to account for observational/residual variance. Phylogeny was controlled for by including a relatedness matrix derived from the phylogenetic tree (using Grafen's method to compute branch lengths) as a random effect in the model. As an index of heterogeneity, we assessed the proportion of the total variance (the total of all variance components in a model) accounted for by a particular random factor (Nakagawa & Santos, 2012). We repeated the same random‐effects model using the conservative data subset described above. We also repeated this model in each taxonomic group (mammals, birds, turtles, squamate reptiles), and in each trait category (mass/size, survival, performance and behavior, physiology, stress response) to obtain taxa‐ and trait‐specific meta‐analytic means. In the turtle data subset, only two species were represented (from two studies), so in this model phylogeny was not included, and only study was included as a random term as this covaried with species.

We conducted sensitivity analyses to further test the influence of non‐independence of data points from the same study by calculating model coefficients and confidence intervals derived from robust variance estimation (Hedges et al., 2010). As robust variance estimation did not substantially alter effect sizes (see Section 3), these models are reported in Appendix 4 (A4.1). To test for unequal variances between the compared groups (heteroskedasticity), we repeated the above model replacing Hedges’ *g* with lnCVR (with associated sampling variance). There was no evidence that variance differed between the compared groups (lnCVR meta‐analytic mean −0.02 ± 0.04, 95% CI: −0.09, 0.05; *p* = .53) so we do not report variance results further (see Appendix 4.2 for full model results).

#### 2.1.2 Effects of moderators

We next tested the extent to which our moderators of interest (Table 1) explained variation in glucocorticoid‐mediated maternal effects on offspring traits (Hedges’ *g*). Our global model included the following moderator variables: reproductive mode (oviparous/viviparous); timing of treatment (early/late/throughout development); the age at which the offspring trait was measured (at or around birth [perinatal], as juvenile, at maturity); trait category (size/mass, physiology, behavior/performance, stress response, survival); and maternal manipulation type (ecological stressor, HPA manipulation). These explanatory variables produced a set of candidate models that were then compared using the AICc (the second‐order Akaike information criterion) in the *MuMIN* package (version 1.43.17) (Bartón, 2009), with the lowest AICc value indicating the best model fit. A subset of models was generated by calculating the difference between the AICc value of the best‐fitting model and all other models using a cutoff of 2 AICc as the criterion for inclusion in the subset. The relative importance of each variable (sum of weights) and model‐averaged coefficients (using the zero method, i.e., full average assuming that a variable is included in every model) were then calculated from this model subset (Burnham et al., 2011). We additionally report the top models. Again, we conducted sensitivity analyses by calculating model coefficients and confidence intervals derived from robust variance estimation, both in the global and in the derived models. As robust variance estimation did not substantially alter the top model subset, or effect sizes in derived models (though confidence intervals typically increased), these models are reported in Appendix 4.3.

Because we were also interested in how moderators influenced the overall strength of glucocorticoid‐mediated maternal effects, and because a priori predictions on directionality were not always possible, we additionally estimated the absolute value of Hedges’ *g*, (|*g*|), which we interpret as the “magnitude” of the effect size. Large effect sizes represent instances where prenatally stressed offspring differ substantially from control offspring (in either direction). To do this, we implemented the best model from the model subset described above, using a Bayesian meta‐analytic meta‐regression model in R in the *MCMCglmm* package (version 2.28; Hadfield, 2010), with the same random‐effects structure as previously described. We applied posterior distributions of parameters from Gaussian models to the folded normal distribution to obtain mean estimates and credible intervals for absolute magnitudes (i.e., “analyze and transform” sensu Morrissey, 2016). MCMC chains were run for 510,000 iterations with a 10,000 iteration burn‐in and a thinning interval of 1000. In total across the three chains, we ran 1,500,000 iterations sampling 1500 iterations from the posterior distribution. Credible intervals not overlapping each other and the value zero suggest statistical significance. Bayesian model coefficients are reported in Appendix 4.4.

#### 2.1.3 Reproductive mode in squamate reptiles

To determine whether or not the influence of reproductive mode persisted while controlling for taxonomy, we tested the influence of moderators within a subset of the data containing only squamate reptiles (*N* = 118 traits, *N* = 9 species, *N* = 20 studies). We produced a set of candidate models derived from the same global model and using the same random term structure as described above. Again, calculating coefficients according to robust variance estimation did not alter effect sizes, but increased confidence intervals—this model is reported in Appendix 4.5. We followed the procedure described above and estimated the magnitude of effect sizes (|*g*|) by implementing the best model from the model subset, using Bayesian meta‐analytic meta‐regression models.

## 3 RESULTS

### 3.1 Overall effects of prenatal stress on offspring traits

**FIGURE 1:**
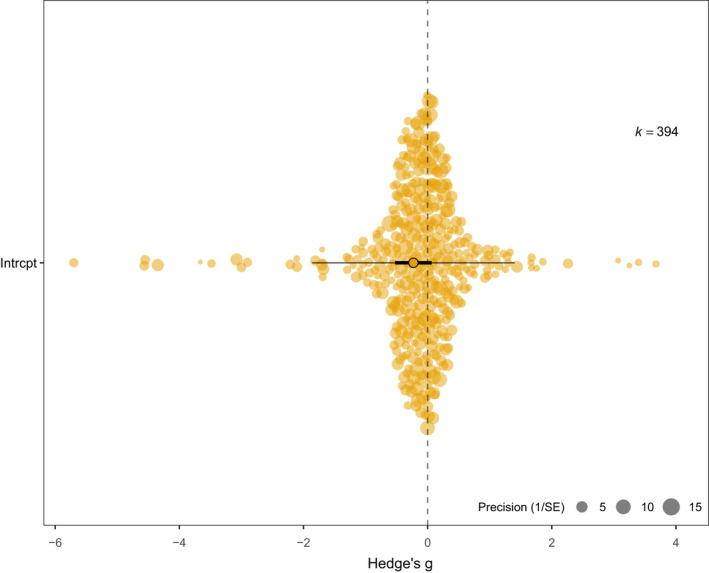
Orchard plot showing overall glucocorticoid‐mediated maternal effects on offspring traits. Position on the *x*‐axis corresponds to effect size value (Hedges’ *g*), with spread on the *y*‐axis based on quasi‐random noise; point size reflects precision, and *k* indicates number of effect sizes (Nakagawa et al., 2021). A dashed vertical line marks 0, that is, no effects, to allow interpretation of direction of effects. Meta‐analytical mean (estimate and error) is shown as a filled, outlined point, and confidence intervals as a black bar

There was an overall negative effect of elevated maternal glucocorticoids/“stress” on offspring traits, though this was not statistically significant (*p* = .13; Table 2; Figure 1). Restricting the dataset to traits where an increase or decrease in value could be more confidently interpreted as having “positive” or”negative” effects did not change this result (*p* = .09; Table 2). Heterogeneity among data was high (*I* ^2^_Total_ = 94.9%, Table 2). Including robust variance estimation (i.e., accounting for covariance of effect sizes and associated sampling variance within studies) did not change effect sizes but did slightly narrow confidence intervals (Appendix 4.1), consistent with heterogeneity being accounted for mostly by residual variance (*I* ^2^_Obser_. = 60.1%) rather than between‐study variance (*I* ^2^_Study_. = 6.53%). The overall effect of maternal stress on mean offspring traits was closer to neutral when modeled separately in each taxonomic group with errors and confidence intervals substantially overlapping zero, suggesting no influence of taxa on variation in prenatal stress effects (Table 2). Random‐effects models in each trait category group also showed average effects closer to neutral (Appendix 4.6). Egger's test of the overall model (all taxonomic groups, using the square root of the inverse combined sample size for each treatment as moderator) showed no significant funnel asymmetry (*p* = .48, Table 2), suggesting a lack of evidence for publication bias or other biases in the overall dataset (e.g., “small sample” or file drawer effects). There was, however, some evidence for funnel asymmetry in the mammalian data subset (Table 2).

**TABLE 2:** Results of random‐effects meta‐analyses models testing the outcomes of glucocorticoid‐mediated maternal effects on offspring traits (Hedges’ *g*) overall, in a data subset where positive/negative outcomes are not subjective (see text for details), and in each taxonomic group

Effect size group	*k*	Meta‐analytic mean ± SE [95% CI]	*I* ^2^ total (%)	*I* ^2^ study (%)	*I* ^2^ species (%)	*I* ^2^ Obs. (%)	*Q* test	Egger's test Est. ± SE [95% CI]
(a) All data	394	−0.23 ± 0.15 [−0.52, 0.06]	94.89	28.27	6.53	60.10	3677	−0.02 ± 0.18 [−0.06, 0.02]
(b) Data subset	233	−0.35 ± 0.20 [−0.75, 0.05]	96.24	50.87	9.86	35.51	2257	−0.03 ± 0.02 [−0.07, 0,02]
(c) Taxonomic groups								
(i) Mammals	110	−0.59 ± 0.61 [−1.80, 0.63]	92.87	4.08	64.43	24.36	524	−0.13 ± 0.06 [−0.26, −0.01]
(ii) Birds	143	−0.05 ± 0.10 [−0.23, 0.14]	73.53	21.82	19.32	32.39	415	0.001 ± 0.02 [−0.02, 0.04]
(iii) Turtles	23	0.04 ± 0.09 [−0.14, 0.22]	100	<0.001	NA	100	27.7	−0.58 ± 0.50 [−1.62, 0.45]
(iv) Squamates	118	−0.40 ± 0.82 [−2.03, 1.23]	99.19	<0.001	69.70	29.41	2657	−0.02 ± 0.04 [−0.09, 0.05]

Note

Model heterogeneity (% *I* ^2^) and the results of Egger's regression tests are also shown.

### 3.2 Effects of moderators

**FIGURE 2:**
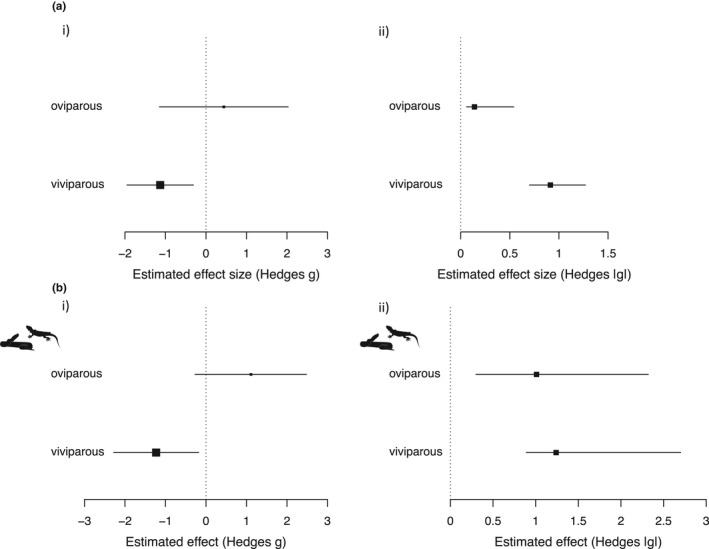
Forest plots showing that there were significantly more negative outcomes of glucocorticoid‐mediated maternal effects in viviparous relative to oviparous species in (ai) the full dataset and (bi) in squamate reptiles. The magnitude (|*g*|, i.e., absolute effects) of prenatal stress was also stronger in (aii) viviparous species overall, though this effect was not apparent in the squamates (bii)

Model comparison using *MuMIn* resulted in a top model subset containing two models: Both included age at measurement and reproductive mode as predictors with the second model also including maternal treatment type (Table 3). The timing of maternal treatment, and the type of trait measured did not appear in any of the top models. The sum of weights, indicating relative variable importance in the top model subset, was as follows: age at measurement 0.99, reproductive mode 0.87, treatment 0.35, timing of treatment 0.16, taxonomic group 0.08, and trait measured 0.03. Model‐averaged parameter estimates (Table 4) and the best model (full results reported in Appendix 4.7) indicate that glucocorticoid‐mediated maternal stress has strong negative effects on offspring in viviparous compared with oviparous species (from best model: *g* viviparous = −1.13, CI 95% = −1.94, −0.31; intercept =0.44, CI 95% = −1.15, 2.03; Figure 2ai). Absolute effects were also considerably stronger in viviparous compared with oviparous species (|*g*| viviparous = 0.91, CI 95% = 0.70, 1.27; oviparous = 0.14, CI 95% = 0.06, 0.54; Figure 2aii). Prenatal stress also had stronger negative effects on average when traits are measured at birth (Table 4; from best model: *g* perinatal = −0.35, CI 95% = −0.54, −0.16; maturity = 0.24, CI 95% = −0.19, 0.67; intercept = 0.44, CI 95% = −1.15, 2.03). Effects on traits measured at birth were also moderately stronger compared with effects on traits measured in the juvenile stage and at maturity, though there was considerable overlap of credible intervals (|*g*| perinatal = 0.74, CI 95% =0.55, 1.29; juvenile = 0.47, CI 95% = 0.28, 1.02; maturity = 0.63, CI 95% = 0.33, 1.24). Maternal treatment type (ecological stressor vs direct HPA manipulation) appeared in the top model subset but was not an important moderator according to averaged estimates.

**TABLE 3:** Subset of top models of moderator effects on effect sizes in (a) all data, and (b) squamate reptiles based on model comparison using AICc with cutoff of Δ2AIC

	Moderator(s)	df	logLik	AICc	Delta	Weight
(a) All data	1 2	7	−473.34	961.0	0.00	0.67
	1 2 3	8	−472.99	962.4	1.39	0.33
(b) Squamates	1 2 3	7	−173.03	361.1	0.00	0.50
	1 2	6	−174.70	362.2	1.09	0.29
	2 3	6	174.99	362.7	1.67	0.22

Note

Term codes: 1—age at measurement; 2—reproductive mode; 3—treatment type.

**TABLE 4:** Results from model comparison testing moderator influence on glucocorticoid‐mediated maternal effects

	Estimate	SE	*z*	*p*
Intercept	0.06	0.15	0.44	.66
Reproductive mode				
Oviparous				
Viviparous	−0.39	0.15	2.62	.01*
Age at offspring measurement				
Juvenile				
Perinatal	−0.34	0.10	3.40	.001**
Maturity	0.12	0.18	0.67	.50
Prenatal treatment				
Ecological stressor				
HPA manipulation	0.05	0.12	0.40	.69

Note

A top model set was derived from a global model containing all moderators using MuMIN. Coefficients from the top model set (<2AICc of best model) were averaged using the zero method, that is, full average to provide overall estimates.

### 3.3 Reproductive mode in squamate reptiles

Model comparison using *MuMIn* resulted in a top model subset containing three models: All three contained reproductive mode (Table 3). The timing of maternal treatment, and the type of trait measured did not appear in any of the top models. The sums of weights were as follows: reproductive mode 0.79, age at measurement 0.67, treatment 0.59, timing of treatment 0.11, and trait measured 0.02. Model‐averaged parameter estimates (Table 5) and the best model (full results reported in Appendix 4.8) indicate that, as in the whole dataset, glucocorticoid‐mediated maternal effects have particularly negative consequences for offspring in viviparous compared with oviparous squamates (from best model: *g* viviparous = −1.23, CI 95% = −2.27, −0.19; intercept =1.13, CI 95% = −0.23, 2.49; Figure 2bi), though the absolute effect of glucocorticoid‐mediated maternal effects was not significantly different between the two groups (|*g*| viviparous = 1.24, CI 95% =0.89 – 2.70; oviparous = 1.01, CI 95% = 0.30–2.32, Figure 2bii). Maternal treatment type (ecological stressor vs direct HPA manipulation) and age at offspring measurement appeared in the top model subset but were not important moderators according to averaged estimates (Table 5).

**TABLE 5:** Results from model comparison testing moderator influence on glucocorticoid‐mediated maternal effects in squamate reptiles

	Estimate	SE	*z*	*p*
Intercept	0.91	0.61	1.49	.14
Reproductive mode				
Oviparous				
Viviparous	−1.13	0.46	2.47	.01*
Age at offspring measurement				
Juvenile				
Perinatal	−0.32	0.24	1.31	.19
Prenatal treatment				
Ecological stressor				
HPA manipulation	−0.38	0.34	1.11	.27

Note

A top model set was derived from a global model containing all moderators using MuMIN. Coefficients from the top model set (<2AICc of best model) were averaged using the zero method, that is, full average to provide overall estimates.

## 4 DISCUSSION

Experimental studies demonstrate that glucocorticoid‐mediated maternal effects can have significant consequences for offspring traits in amniotic vertebrates, but the effects are very variable (e.g., Agrawal, 2001; Cottrell, 2009; Love et al., 2013; Seckl, 2004; Sheriff & Love, 2013; Storm & Lima, 2010; Welberg & Seckl, 2001). Here, we demonstrate that these effects are stronger and generally likely more negative in viviparous compared with oviparous species. Importantly, the difference between viviparous and oviparous species was evident across both amniotes as a whole (mammals and viviparous squamates vs. birds, turtles and oviparous squamates) and within squamate reptiles. Together, this provides strong evidence that prolonged physiological association between embryo and mother is a major determinant of the strength of glucocorticoid‐mediated maternal effects on offspring phenotype, suggesting hitherto undervalued consequences associated with the evolution of viviparity.

There was substantial variation in the magnitude and direction of trait responses to glucocorticoid‐mediated maternal stress. The fitness consequences of these responses are difficult to interpret on the basis of phenotypic variation alone since they will depend on the local environment, and the link to fitness for many traits is unclear (e.g., activity or variation in certain behaviors such as grooming, or movement). Thus, it is possible that the variation in effect sizes reflect both maladaptive outcomes and adaptive potential of glucocorticoid‐mediated maternal effects. Together with the high degree of effect size heterogeneity in our results (*I* ^2^_Total_ = 94.9%), this suggests that glucocorticoid‐mediated maternal effects are very dependent on the developmental biology of the species, ecological context, and the function of the target traits. This is not surprising, but it cautions against interpretation of phenotypic effects in terms of adaptive value or negative impact of maternal/prenatal stress without substantial additional evidence. Nevertheless, it is noticeable that there was a bias toward phenotypic effects that are more likely to be detrimental based on life history theory, a result that held up for a conservative inclusion of traits for which fitness effects may be more reliably predicted (e.g., growth and body size, for which a negative effect is likely to indeed be “negative” for offspring fitness; Roff, 1993). These results are in line with a meta‐analysis of developmental stress across a wider range of taxonomic groups (including domesticated and laboratory strains), where there was an overall negative effect of more broadly defined developmental stress on animal phenotype or performance (posterior mean effect: |*d*| = −0.51; Eyck et al., 2019). However, as this study (Eyck et al., 2019) did not include most of the studies in our dataset (but included many laboratory studies of rodents), it is difficult to assess whether the effects of prenatal glucocorticoids in amniotes are comparably weak in comparison with other forms of developmental stress in animals.

Our results suggest that glucocorticoid‐mediated maternal effects are stronger and generally likely to be more negative than positive in mammals and viviparous squamate reptiles compared with birds, turtles, and oviparous squamates. That the effect of reproductive mode was substantially stronger and more consistent than other life history characters, including within non‐avian reptiles, suggests that these prenatal effects are strongly shaped by opportunity. Indeed, the longer period of interaction between mothers and developing offspring in viviparous species (Blackburn, 1999; Schatten & Constantinescu, 2008) may result in a more consistent exposure to glucocorticoids (Meaney et al., 2007). Viviparity also provides a greater opportunity for other, indirect, effects of elevated maternal stress or glucocorticoids on offspring. For example, maternal stress has been shown to influence female body weight and condition (De Vos et al., 1995; Klein, 2015); food intake (Cote et al., 2006; Osborne, 2015); rates of metabolism (Haase et al., 2016); immune function (McCormick et al., 2014); and the capacity of the placenta to transport key nutrients and flow‐limited substrates (e.g., carbon dioxide and oxygen (Mairesse et al., 2007; Myatt, 2006)). All of these changes have the potential to mediate offspring development, and ultimately phenotype. For example, reduced maternal investment as a result of gestational stress is linked to reduced offspring birth weight (Berghänel et al., 2017). Note that here, a “negative” effect on an offspring trait may be adaptive for the mother (e.g., reduced maternal investment that promotes her survival and future reproduction; Roff, 1993). In contrast, although it has been established that glucocorticoids and other steroid hormones are passed to eggs by mothers in oviparous species, the levels are typically low (Groothuis et al., 2005, 2019; Hayward & Wingfield, 2004). Furthermore, steroid hormones that are deposited in the egg can be metabolized early in life, resulting in rapid (and permanent) declines in hormone concentrations during later developmental stages (Paitz & Bowden, 2009; Paitz et al., 2011; Vassallo et al., 2014). Thus, the level and duration of glucocorticoid exposure during development should be limited in oviparous species relative to viviparous species.

That the strength and direction of glucocorticoid‐mediated maternal effects in viviparous squamates appears similar to mammals, whereas oviparous squamates were more similar to birds, has some interesting implications for the role of complex placentation in mediating stress responses. In squamates, significant placentotrophy is restricted to skinks (Scincidae), with the majority of the 800+ viviparous squamates being lecithotrophic (Blackburn, 2006; Thompson & Speake, 2006). In the latter, embryonic nutrition is primarily derived from an ovulated yolk, while the placental membrane mostly functions as a medium for gas exchange and some water and nutrient transport (Blackburn, 1992; Thompson & Speake, 2006; Van Dyke et al., 2014). In our dataset, the majority of species are lecithotrophic (82 of the 87 effect sizes associated with viviparous reptiles; only one study representing a highly placentrophic species, *Carinascincus ocellatus*; Thompson et al., 2001). While no specific causal mechanisms of prenatal stress can be excluded on the basis of these results (see above), the fact that we see strong effects in viviparous reptiles suggests that biologically significant transfer of glucocorticoids from mother to offspring occurs in species even with morphologically simple chorioallantoic placentae (Painter et al., 2002).

A prolonged period of feto‐maternal interaction is thought to result in a suite of important consequences for subsequent evolution, for example, by increasing the potential for parental–offspring and intragenomic conflict (Haig, 2014; Zeh & Zeh, 2000). Our results suggest that disruption of endocrine regulation could be a further physiological cost of the evolution of viviparity (Painter et al., 2002; Uller & Olsson, 2006). However, an extended period of association between mothers and offspring, and the capacity for regulatory control via the placenta, could also enable more precise adjustment of offspring phenotype to “match” local conditions at birth (Uller, 2008). Additionally, “negative” effects on traits (such as reduced body size) could be adaptive in some contexts (e.g., under high predation pressure, Langerhans, 2009; Riesch et al., 2013). The context specificity of fitness outcomes of glucocorticoid‐mediated maternal effects has rarely been tested, but is crucial to infer whether maternal effects on offspring phenotype are likely to be adaptive (Engqvist & Reinhold, 2016; Uller et al., 2013).

While the evolution of complex placentation could be accompanied by more sophisticated mechanisms that regulate interactions between mothers and offspring (Blackburn, 1992, 1998), the effects in viviparous squamates and mammals were comparable for prenatal stress effects. The ubiquity and necessity of glucocorticoids across metabolic and developmental processes (Rose & Herzig, 2013; Turkay et al., 2012), and the role of maternal‐fetal glucocorticoid exchange in pregnancy maintenance and parturition (Chida et al., 2011; Cole et al., 1995; Pepe & Albrecht, 1995), likely selects against complete placental buffering in species with complex placentas, potentially explaining the similarity between squamates and mammals. This may also explain why we found variation between egg‐laying and live‐bearing species in prenatal stress effects driven by glucocorticoids, while there is no general effect of reproductive mode on the strength of maternal effects more generally (Moore et al., 2019).

The phenotypic outcomes of glucocorticoid‐mediated maternal effects appeared to be most strongly negative when offspring were measured at or close to birth, while traits measured as juveniles or in adulthood were generally neutral. This decline in effect strength over ontogeny matches previous work on developmental stress effects (Berghänel et al., 2017), as well as maternal effects generally (Bernardo, 1996; DiBattista et al., 2009; Houde et al., 2013; Lindholm et al., 2006; Wilson & Réale, 2006). For example, a meta‐analysis of 770 estimates across 116 studies showed that maternal effects had a greater influence over juvenile relative to adult traits (though maternal effects still explained some adult phenotypic variation: Moore et al., 2019). Even where glucocorticoid‐mediated maternal effects are adaptive (Sheriff & Love, 2013), parental information becomes increasingly less reliable with time and individuals acquire information based on their own experience (English et al., 2015; McNamara et al., 2016).

Lastly, our study highlights some useful points about how prenatal stress and its consequences are studied. The effects on offspring phenotype were, on the whole, stronger/more negative when the HPA axis was directly manipulated rather than when mothers were exposed to a “natural” stressor. Manipulating only one aspect of the stress response may be more effective, but it may also provide an unrealistic reflection of how maternal stress mediates prenatal development in the wild. For example, glucocorticoid manipulations may exceed ecologically relevant ranges, or phenotypic effects may be caused by pleiotropic effects of glucocorticoids unrelated to the stress responses (MacDougall‐Shackleton et al., 2019; Sopinka et al., 2015). As a result, manipulations of HPA axis may overestimate the effects of environmental stressors on offspring (since the effects of “natural” stress were weaker on average). On the contrary, it is notable that only 14 studies conformed to our criteria that a maternal “stressor” treatment had to demonstrate some effect on maternal stress physiology. Moving forward, a better integration of physiological data and ecological context is needed to establish the phenotypic effects and functional significance of prenatal stress.

## CONFLICT OF INTEREST

The authors declare no conflicts of interest.

## AUTHOR CONTRIBUTIONS

**Kirsty J. MacLeod:** Conceptualization (equal); Data curation (lead); Formal analysis (lead); Funding acquisition (lead); Investigation (lead); Methodology (lead); Project administration (lead); Visualization (lead); Writing‐original draft (lead); Writing‐review & editing (lead). **Geoffrey While:** Conceptualization (equal); Funding acquisition (supporting); Methodology (equal); Visualization (supporting); Writing‐original draft (supporting); Writing‐review & editing (equal). **Tobias Uller:** Conceptualization (equal); Formal analysis (supporting); Funding acquisition (supporting); Methodology (equal); Project administration (supporting); Visualization (supporting); Writing‐original draft (supporting); Writing‐review & editing (equal).

## Data Availability

All data and analysis code are available at 10.5281/zenodo.5710355.

## APPENDIX 1

### Preferred reporting items for systematic reviews and meta‐analyses

*Search term combinations used*: transgenerational glucocorticoid/corti*; anticipatory maternal effect; environmental matching; prenatal stress/glucocorticoid/corti*; transgenerational stress mammal/bird/reptile; maternal corti* bird/mammal/reptile; maternal stress wild rodent; maternal glucocorti*; maternal stress effect mammal ‐human ‐rat ‐domestic ‐depression; maternal stress effect bird/reptile.

## APPENDIX 2

### Trait categories and specific traits in each

Traits for which effect size signs were changed (i.e., where an increase in a trait value reflected a negative/detrimental functional outcome) are italicized. Traits included in the restricted subset are marked with an X.

**Table:**

Trait category	Specific trait	Included in subset	Studies appeared in
Performance/behavior	Number times of righted	X	1
	*Fastest time righting*	X	1
	*Righting response time*	X	2
	Tactile response (0/1)		4
	Distance traveled		4
	Time basking/under shelter		4, 18
	Exploratory rate		7
	Thigmotaxis rate		7
	Sprint speed	X	9, 10, 16
	Escape behavior		12, 47
	Dispersal rate/probability	X	13, 19, 20, 21
	Dispersal distance		31
	Swimming endurance	X	14
	*Latency to resume normal behavior after predator cue*		14
	Time spent moving		18
	Likelihood of seeking refuge		21
	Time spent scratching in new environment		21
	Time active in new environment		21
	Begging duration/loudness/max frequency/rate		24, 26, 35, 36
	*Reversal‐to‐prone response*	X	35
	Activity		24
	*Tonic immobility in response to restraint*		26
	Open field trial responses		37
	Maze test responses		37
	Time spent alert/foraging/exploring/hiding		40
	Contribution to care activities		46
Survival	Survival to time point/life history event (e.g., dispersal)	X	1, 6, 17, 22, 46
Mass/size	Mass	X	1, 3, 4, 5, 6, 9, 10, 11, 13, 14, 16, 22, 23, 24, 25, 26, 29, 30, 31, 32, 33, 34, 36, 37, 39, 40, 41, 42, 43, 44, 46
	Total/body length	X	1, 2, 4, 5, 6, 9, 10, 11, 12, 13, 14, 15, 16, 31, 33, 38, 40, 43
	Feather length	X	44
	Tail length	X	42
	Limb/wing length	X	4, 10
	Growth (general, of specific body part)	X	3, 4, 22, 23, 26, 29, 31, 42, 47 8, 9, 10, 17, 25, 30, 32, 39, 40
	Lean dry muscle mass	X	27
	Body condition metric	X	5, 6, 9, 13, 17, 21, 28, 38, 44
Stress response	PHA response (stress)	X	3, 25, 29
	*CORT levels (baseline/stress‐induced/CORT in recovery phase)*	X	4, 22, 24, 28, 34, 37, 39, 40, 45, 46, 47
	CBG capacity		24
	Stress‐induced blood glucose		37
	ACTH levels		4, 40
Physiology	Hematocrit		3
	*fluctuating asymmetry*		15
	Antioxidant capacity		23
	Oxidative status		23
	Heterophil/lymphocyte ratio and counts		24, 37, 41
	Breathing rate		24
	Antibody response/T‐cell response		26
	Muscle water/fat content		27
	Wing loading		27
	Enzyme activity		27
	Antibody titer		37, 41
	Immune swelling response		37
	Blood glucose		37
	Organ mass to body mass ratio		40, 41, 44
	fosB mRNA expression/protein levels		40
	Hormone levels		44
	Telomere length/telomerase activity		45, 48
	Mitochondrial content		47
	DNA methylation		47

1. Polich, R., Bodensteiner, B. L., Adams, C. I. M., & Janzen, F. J. (2018). Effects of augmented corticosterone in painted turtle eggs on offspring development and behavior. *Physiology and Behavior*, *183*, 1–9.

2. Carter, A. W., Paitz, R. T., McGhee, K. E., & Bowden, R. M. (2016). Turtle hatchlings show behavioral types that are robust to developmental manipulations. *Physiology and Behavior*, *155*, 46–55.

3. Strange, M. S., Bowden, R. M., Thompson, C. F., & Sakaluk, S. K. (2016). Pre‐ and postnatal effects of corticosterone on fitness‐related traits and the timing of endogenous corticosterone production in a songbird. *Journal of Experimental Zoology. Part A, Ecological Genetics and Physiology*, *325*(6), 347–359.

4. Ensminger, D. C., Langkilde, T., Owen, D. A. S., MacLeod, K. J., & Sheriff, M. J. (2018). Maternal stress alters the phenotype of the mother, her eggs and her offspring in a wild‐caught lizard. *Journal of Animal Ecology*, *87*(6), 1685–1697.

5. Owen, D. A. S., Sheriff, M. J., Engler, H. I., & Langkilde, T. (2018). Sex‐dependent effects of maternal stress: stressed moms invest less in sons than daughters. *Journal of Experimental Zoology Part A: Ecological and Integrative Physiology*, *329*(6–7), 317–322.

6. Dupoué, A., Le Galliard, J. F., Josserand, R., DeNardo, D. F., Decencière, B., Agostini, S., Haussy, C., & Meylan, S. (2018). Water restriction causes an intergenerational trade‐off and delayed mother–offspring conflict in a viviparous lizard. *Functional Ecology*, *32*(3), 676–686.

7. Rozen‐Rechels, D., Dupoué, A., Meylan, S., Decencière, B., Guingand, S., & Le Galliard, J. F. (2018). Water restriction in viviparous lizards causes transgenerational effects on behavioral anxiety and immediate effects on exploration behavior. *Behavioral Ecology and Sociobiology*, *72*(2).

8. Dupoué, A., Angelier, F., Brischoux, F., DeNardo, D. F., Trouvé, C., Parenteau, C., & Lourdais, O. (2016). Water deprivation increases maternal corticosterone levels and enhances offspring growth in the snake Vipera Aspis. *The Journal of Experimental Biology*, *219*(5), 658–667.

9. Cadby, C. D., Jones, S. M., & Wapstra, E. (2010). Are increased concentrations of maternal corticosterone adaptive to offspring? A test using a placentotrophic lizard. *Functional Ecology*, *24*(2), 409–416.

10. Warner, D. A., Rajkumar, R. S., & Shine, R. (2009). Corticosterone exposure during embryonic development affects offspring growth & sex ratios in opposing directions in two lizard species with environmental sex determination. *Physiological & Biochemical Zoology*, *82*(4), 363–371.

11. Uller, T., Hollander, J., Astheimer, L., & Olsson, M. (2009). Sex‐specific developmental plasticity in response to yolk corticosterone in an oviparous lizard. *Journal of Experimental Biology*, *212*(8), 1087–1091.

12. Robert, K. A., Vleck, C., & Bronikowski, A. M. (2009). The effects of maternal corticosterone levels on offspring behavior in fast‐ and slow‐growth garter snakes (*Thamnophis elegans*). *Hormones and Behavior*, *55*(1), 24–32.

13. Vercken, E., de Fraipont, M., Dufty, A. M., & Clobert, J. (2007). Mother's timing and duration of corticosterone exposure modulate offspring size and natal dispersal in the common lizard (*Lacerta vivipara*). *Hormones and Behavior*, *51*(3), 379–386.

14. Uller, T., & Olsson, M. (2006). Direct exposure to corticosterone during embryonic development influences behaviour in an ovoviviparous lizard. *Ethology*, *112*(4), 390–397.

15. Uller, T., Meylan, S., de Fraipont, M., & Clobert, J. (2005). Is sexual dimorphism affected by the combined action of prenatal stress and sex ratio? *Journal of Experimental Zoology Part A: Comparative Experimental Biology*, *303*(12), 1110–1114.

16. Preest, M. R., Cree, A., & Tyrrell, C. L. (2005). ACTH‐induced stress response during pregnancy in a viviparous gecko: No observed effect on offspring quality. *Journal of Experimental Zoology Part A: Comparative Experimental Biology*, *303*(9), 823–835.

17. Meylan, S., & Clobert, J. (2005). Is corticosterone‐mediated phenotype development adaptive? Maternal corticosterone treatment enhances survival in male lizards. *Hormones and Behavior*, *48*(1), 44–52.

18. Belliure, J., Meylan, S., & Clobert, J. (2004). Prenatal and postnatal effects of corticosterone on behavior in juveniles of the common lizard, Lacerta Vivipara. *Journal of Experimental Zoology*, *301A*, 401–410.

19. Meylan, S., de Fraipont, M., & Clobert , J. (2004). Maternal size and stress and offspring philopatry: An experimental study in the common lizard (Lacerta Vivipara). *Écoscience*, *11*(1), 123–129.

20. Meylan, S., Belliure, J., Clobert, J., & de Fraipont, M. (2002). Stress and body condition as prenatal and postnatal determinants of dispersal in the common lizard (Lacerta Vivipara). *Hormones and Behavior*, *42*, 319–326.

21. De Fraipont, M., Clobert, J., John Alder, H., & Meylan, S. (2003). Increased pre‐natal maternal corticosterone promotes philopatry of offspring in common lizards Lacerta Vivipara. *Journal of Animal Ecology*, *69*(3), 404–413.

22. Weber, B. M., Bowers, K. E., Terrell, K. A., Falcone, J. F., Thompson, C. F., & Sakaluk, S. K. (2018). Pre‐ and postnatal effects of experimentally manipulated maternal corticosterone on growth, stress reactivity and survival of nestling house Wrens. *Functional Ecology*, *32*(8), 1995–2007.

23. Possenti, C. D., Secomandi, S., Schiavon, A., Caprioli, M., Rubolini, D., Romano, A., Saino, N., & Parolini, M. (2018). Independent and combined effects of egg pro‐ and anti‐oxidants on gull chick phenotype. *The Journal of Experimental Biology*. jeb.174300‐jeb.174300.

24. Tilgar, V., Mägi, M., Lind, M., Lodjak, J., Moks, K., & Mänd, R. (2016). Acute embryonic exposure to corticosterone alters physiology, behaviour and growth in nestlings of a wild passerine. *Hormones and Behavior*, *84*, 111–120.

25. Love O. P., Chin, E. H., Wynne‐Edwards, K. E., & Williams, T. D. (2017). Stress hormones: A link between maternal condition and sex‐biased reproductive investment. *The American Naturalist*, *166*(6), 751–751.

26. Rubolini, D., Romano, M., Boncoraglio, G., Ferrari, R. P., Martinelli, R., Galeotti, P., Fasola, M., & Saino, N. (2005). Effects of elevated egg corticosterone levels on behavior, growth, and immunity of yellow‐legged gull (*Larus michahellis*) chicks. *Hormones and Behavior*, *47*(5), 592–605.

27. Chin, E. H., Love, O. P., Verspoor, J. J., Williams, T. D., Rowley, K., & Burness, G. (2009). Juveniles exposed to embryonic corticosterone have enhanced flight performance. *Proceedings of the Royal Society B: Biological Sciences*, *276*(1656), 499–505.

28. Love, O. P., & Williams, T. D. (2008). Plasticity in the adrenocortical response of a free‐living vertebrate: The role of pre‐ and post‐natal developmental stress. *Hormones and Behavior*, *54*(4), 496–505.

29. Love, O. P., & Williams. T. D. (2008). The adaptive value of stress‐induced phenotypes: Effects of maternally derived corticosterone on sex‐biased investment, cost of reproduction, and maternal fitness. *The American Naturalist*, *172*(4), E135–E149.

30. Henderson, L. J., Evans, N. P., Heidinger, B. J., Adams, A., & Arnold, K. E. (2014). Maternal condition but not corticosterone is linked to offspring sex ratio in a passerine bird. *PLoS ONE*, *9*(10), 1–10.

31. Coslovsky, M., & Richner, H. (2011). Predation risk affects offspring growth via maternal effects. *Functional Ecology*, *25*(4), 878–888.

32. Dantzer, B., Newman, A. E. M., Boonstra, R., Palme, R., Boutin, S., Humphries, M. M., & McAdam, A. G. (2013). Density triggers maternal hormones that increase adaptive offspring growth in a wild mammal. *Science*, *1215*, 1215–1218.

33. Sheriff, M. J., Krebs, C. J., & Boonstra, R. (2009). The sensitive hare: Sublethal effects of predator stress on reproduction in snowshoe hares. *The Journal of Animal Ecology*, *78*(6), 1249–1258.

34. Bian, J. H., Du, S. Y., Wu, Y., Cao, Y. F., Nue, X. H., & You, Z. B. (2015). Maternal effects & population regulation: Maternal density‐induced reproduction suppression impairs offspring capacity in response to immediate environment in root voles microtus oeconomus. *Journal of Animal Ecology*, *84*(2), 326–336.

35. Possenti, C. D., Parolini, M., Romano, A., Caprioli, M., Rubolini, D., & Saino, N. (2018). Effect of yolk corticosterone on begging in the yellow‐legged gull. *Hormones and Behavior*, *97*(2017), 121–127.

36. Bowers, K. E., Bowden, R. M., Thompson, C. F., & Sakaluk, S. K. (2016). Elevated corticosterone during egg production elicits increased maternal investment and promotes nestling growth in a wild songbird. *Hormones and Behavior*, *83*, 6–13.

37. Brachetta, V., Schliech, C. E., Cutrera, A. P., Merlo, J. L., Kittlein, M. J., & Zenuto, R. R. (2018). Prenatal predatory stress in a wild species of subterranean rodent: Do ecological stressors always have a negative effect on the offspring? *Developmental Psychobiology*, *60*(5), 567–581.

38. Meylan, S., Haussy, C., & Voituron, Y. (2010). Physiological actions of corticosterone and its modulation by an immune challenge in reptiles. *General and Comparative Endocrinology*, *169*(2), 158–166.

39. Bian J., Wu, Y., & Liu, J. (2005). Effect of predator‐induced maternal stress during gestation on growth in root voles Microtus Oeconomus. *Acta Theriologica*, *50*(4), 473–482.

40. Gu, C., Wang, W., Ding, X., Yang, S., Wang, A., Yin, B., & Wei, W . (2018). Effects of maternal stress induced by predator odors during gestation on behavioral and physiological responses of offspring in Brandt's Vole (*Lasiopodomys brandtii*). *Integrative Zoology*, 13(6), 723–734.

41. Yan, W., Jianghui, B., & Yifan, C. A. O. (2008). 围栏条件下母体社群应激对根田鼠 子代免疫力的影响 吴雁 *28*, 250–259.

42. Saino, N., Romano, M., Ferrari, R. P., Martinelli, R., & Møller, A. P. (2005). Stressed mothers lay eggs with high corticosterone levels which produce low‐quality offspring. *J. Exp. Zool*, *303*, 998–1006.

43. Lancaster, L. T., Hazard, L. C., Clobert, J., & Sinervo, B. R . (2008). Corticosterone manipulation reveals differences in hierarchical organization of multidimensional reproductive trade‐offs in r‐Strategist and K‐Strategist females. *Journal of Evolutionary Biology*, *21*(2), 556–565.

44. Gu, C., Liu, Y., Huang, Y., Wang, A., Yin, B., & Wei, W. (2020). Effects of predator‐induced stress during pregnancy on reproductive output and offspring quality in Brandt's voles (*Lasiopodomys brandtii*). *European Journal of Wildlife Research*, *66*(1), 14.

45. Noguera, J. C., & Velando, A. (2019). Reduced telomere length in embryos exposed to predator cues. *Journal of Experimental Biology*, jeb.216176.

46. Dantzer, B., Dubuc, C., Goncalves, I. B., Cram, D. L., Bennett, N. C., Ganswindt, A., Heistermann, M., Duncan, C., Gaynor, D., & Clutton‐Brock, T. H. (2019). The development of individual differences in cooperative behaviour: Maternal glucocorticoid hormones alter helping behaviour of offspring in wild Meerkats. *Philosophical Transactions of the Royal Society B: Biological Sciences*, *374*(1770), 20180117.

47. Noguera, J. C., & Velando, A. (2019). Bird embryos perceive vibratory cues of predation risk from clutch mates. *Nature Ecology & Evolution*, 3(8), 1225–1232.

48. Noguera, J. C., Silva, A., & Velando, A. (2020). Egg corticosterone can stimulate telomerase activity and promote longer telomeres during embryo development. *Molecular Ecology*. mec.15694

## APPENDIX 3

### Phylogenetic tree

Created using a synthetic super‐tree from the Open Tree of Life database (Hinchliff et al., 2015), accessed and pruned through the R package *rotl* (Michonneau et al., 2016). Major taxonomic groups are separated by color (labeled on right). Viviparous species are indicated with open circles as nodes; oviparous species are indicated with filled circles as nodes.

## APPENDIX 4

### Results of statistical models

4.1. Results of random‐effects meta‐analysis models testing the effect of prenatal stress on (a) mean offspring traits (Hedges’ *g*) and (b) using sensitivity analyses, that is, reporting robust variance estimates with study ID as a cluster

**Table:**

	Est.	SE	*t*	*p*	ci.lb	ci.ub
*(a) Random‐effects models, no sensitivity analysis*						
Random effects—all taxa	−0.23	0.15	−1.54	.13	−0.52	0.06
Mammals	−0.59	0.61	−0.96	.34	−1.80	0.63
Birds	−0.05	0.09	−0.48	.63	−0.23	0.14
Turtles	0.04	0.09	0.49	.63	−0.14	0.22
Squamates	−0.40	0.83	−0.48	.63	−2.03	1.23
*(b) Random‐effects models with robust variance estimation*						
Random effects—all taxa (48 clusters)	−0.23	0.09	−2.40	.02	−0.42	−0.04
Mammals (9 clusters)	−0.59	0.49	−1.21	.26	−1.71	0.53
Birds (17 clusters)	−0.05	0.06	−0.82	.43	−0.16	0.07
Turtles (2 clusters)	0.04	0.03	1.47	.38	−0.32	0.41
Squamates (20 clusters)	−0.40	0.33	−1.23	.23	−1.08	0.28

4.2. Meta‐analysis of variance (lnCVR)

**Table:**

Effect size	*k*	Meta‐analytic mean ± SE [95% CI]	*I* ^2^ total (%)	*I* ^2^ study (%)	*I* ^2^ species (%)	*I* ^2^ obser. (%)	*Q* test	Egger's test Est. ± SE [95% CI]
lnCVR	382	−0.02 ± 0.04 [−0.09, 0.05]	92.41	12.78	<0.001	79.63	4102	0.02 ± 0.01 [0.004, 0.04]
Mammals	110	0.02 ± 0.11 [−21, 0.24]	81.96	35.54	<0.001	46.25	666	0.001 ± 0.04 [−0.08, 0.10]
Birds	141	0.003 ± 0.11 [−0.21, 0.21]	87.59	9.72	32.41	45.46	980	0.03 ± 0.01 [0.01, 0.05]
Turtles	23	−0.24 ± 0.15 [−0.55, 0.07]	89.27	<0.001	<0.001	89.27	170	1.94 ± 0.84 [0.20, 3.69]
Squamates	108	−0.02 ± 0.06 [−0.15, 0.10]	96.12	1.56	<0.001	94.56	2243	0.01 ± 0.02 [−0.03, 0.05]

4.3. Best model testing moderator influence on prenatal stress effects according to model comparison, with coefficients derived from robust variance estimation

**Table:**

	Estimate	SE	*t*	*p*	ci.lb	ci.ub
Intercept	0.44	0.43	1.04	.30	−0.41	1.30
Reproductive mode						
Oviparous						
Viviparous	−1.13	0.70	−1.62	.11	−2.53	0.28
Age at offspring measurement						
Juvenile						
Perinatal	−0.35	0.13	−2.76	.01*	−0.60	−0.09
Maturity	0.24	0.24	1.01	.32	−0.24	0.72

4.4. Bayesian models—analyzing the magnitude of effects in models of interest.

(a) All data (*N* = 394)

Best model from model comparison (MuMIn) = *yi* ~ repro mode + age at measurement.

Table showing estimates and confidence intervals (95%) from MCMCglmm—Hedges’ *g*, and magnitude of effects |*g*| derived by applying the folded normal distribution sensu Morrisey “analyze‐then‐transform” (Morrissey, 2016).

**Table:**

Moderator	*g*	|*g*|
Reproductive mode		
Oviparous	0.09 (−0.29, 0.52)	0.14 (0.06, 0.54)
Viviparous	−0.51 (−1.04, −0.09)	0.91 (0.70, 1.27)
Age at measurement		
Juvenile	0.09 (−0.29, 0.52)	0.47 (0.28, 1.02)
Perinatal	−0.23 (−0.36, −0.11)	0.74 (0.55, 1.29)
Maturity	0.20 (−0.09, 0.54)	0.63 (0.33, 1.24)

(b) Squamates only (*N* = 128)

Best model from model comparison (MuMIn) = *yi* ~ repro mode + age at measurement + treatment type.

Table showing estimates and confidence intervals (95%) from MCMCglmm—Hedges’ *g*, and magnitude of effects |*g*| derived by applying the folded normal distribution sensu Morrisey “analyze‐then‐transform” (Morrissey, 2016).

**Table:**

Moderator	*g*	|*g*|
Reproductive mode		
Oviparous	0.88 (−0.48, 2.03)	1.01 (0.30, 2.32)
Viviparous	−0.91 (−2.15, −0.21)	1.24 (0.89, 2.70)
Age at measurement		
Juvenile	0.88 (−0.48, 2.03)	1.23 (0.79, 3.97)
Perinatal	−0.44 (−0.77, −0.12)	1.49 (0.75, 3.04)
Treatment type		
Ecological stressor	0.88 (−0.48, 2.03)	0.74 (0.27, 4.61)
HPA manipulation	−0.50 (−1.19, 0.22)	1.42 (0.95, 5.40)

4.5. Results from model comparison testing moderator influence on prenatal stress effects in squamates, with coefficients derived from robust variance estimation. A top model set was derived from a global model containing all moderators using MuMIN. Coefficients from the top model set (<2AICc of best model) were averaged (a) to provide overall estimates. The best model is reported (b)

**Table:**

	Estimate	SE	*t*	*p*	ci.lb	ci.ub
Intercept	1.13	0.72	1.57	.14	−0.39	2.65
Reproductive mode						
Oviparous						
Viviparous	−1.23	0.84	−1.47	.16	−3.00	0.54
Age at offspring measurement						
Juvenile						
Perinatal	−0.39	0.22	1.77	.10	−0.85	0.07
Prenatal treatment						
Ecological stressor						
HPA manipulation	−0.51	0.37	−1.36	.19	−1.30	0.28

4.6. Random‐effects models in each trait category group showing average effects (meta‐analytic means). Heterogeneity and overall test of moderator (*Q* test) also reported

**Table:**

Effect size	*k*	Meta‐analytic mean ± SE [95% CI]	*I* ^2^ total (%)	*I* ^2^ study (%)	*I* ^2^ species (%)	*I* ^2^ Obs. (%)	*Q* test
All data	394	−0.23 ± 0.15 [−0.52, 0.06]	94.89	28.27	6.53	60.10	3677
Trait categories							
(i) Mass_size	197	−0.34 ± 0.21 [−0.75, 0.08]	96.72	56.98	9.96	29.78	1999.48
(ii) Physiology	66	−0.01 ± 0.08 [−0.18, 0.15]	82.40	<0.001	<0.001	82.40	224.17
(iii) Perform_behav	79	−0.20 ± 0.12 [−0.44, 0.04]	94.98	<1	<0.001	94.34	1232.55
(iv) Stress_response	37	−0.11 ± 0.28 [−0.68, 0.45]	87.53	46.76	30.89	9.89	178.91
(v) Survival	15	−0.21 ± 0.18 [−0.59, 0.17]	64.67	26.17	38.49	<0.001	22.79

4.7 Best model derived from model comparison testing moderator influence on glucocorticoid‐mediated maternal effects

**Table:**

	Estimate	SE	*t*	*p*	ci.lb	ci.ub
Intercept	0.44	0.80	0.54	.59	−1.15	2.03
Reproductive mode						
Oviparous						
Viviparous	−1.13	0.42	−2.71	.007**	−1.94	−0.31
Age at offspring measurement						
Juvenile						
Perinatal	−0.35	0.10	−3.54	.0004**	−0.54	−0.16
Maturity	0.24	0.22	1.10	.27	−0.19	0.67

4.8 Best model derived from model comparison testing moderator influence on glucocorticoid‐mediated maternal effects in squamate reptiles only

**Table:**

	Estimate	SE	*t*	*p*	ci.lb	ci.ub
(a) Best model						
Intercept	1.13	0.69	1.64	.10	−0.23	2.49
Reproductive mode						
Oviparous						
Viviparous	−1.23	0.52	−2.34	.02*	−2.27	−0.19
Age at offspring measurement						
Juvenile						
Perinatal	−0.39	0.20	1.94	.06	−0.78	0.01
Prenatal treatment						
Ecological stressor						
HPA manipulation	−0.51	0.29	−1.76	.08	−1.08	0.06
